# Epigenetic Manipulation Facilitates the Generation of Skeletal Muscle Cells from Pluripotent Stem Cells

**DOI:** 10.1155/2017/7215010

**Published:** 2017-04-09

**Authors:** Tomohiko Akiyama, Shunichi Wakabayashi, Atsumi Soma, Saeko Sato, Yuhki Nakatake, Mayumi Oda, Miyako Murakami, Miki Sakota, Nana Chikazawa-Nohtomi, Shigeru B. H. Ko, Minoru S. H. Ko

**Affiliations:** Department of Systems Medicine, Keio University School of Medicine, Tokyo 160-8582, Japan

## Abstract

Human pluripotent stem cells (hPSCs) have the capacity to differentiate into essentially all cell types in the body. Such differentiation can be directed to specific cell types by appropriate cell culture conditions or overexpressing lineage-defining transcription factors (TFs). Especially, for the activation of myogenic program, early studies have shown the effectiveness of enforced expression of TFs associated with myogenic differentiation, such as PAX7 and MYOD1. However, the efficiency of direct differentiation was rather low, most likely due to chromatin features unique to hPSCs, which hinder the access of TFs to genes involved in muscle differentiation. Indeed, recent studies have demonstrated that ectopic expression of epigenetic-modifying factors such as a histone demethylase and an ATP-dependent remodeling factor significantly enhances myogenic differentiation from hPSCs. In this article, we review the recent progress for in vitro generation of skeletal muscles from hPSCs through forced epigenetic and transcriptional manipulation.

## 1. Introduction

The characteristics of cells are principally determined by patterns of gene expression. During the course of development, the generation of various cell types consisting of our body are driven by the dynamic alteration of gene expression patterns. Human pluripotent stem cells (hPSCs) such as embryonic stem cells (ESCs) [1] and induced pluripotent stem cells (iPSCs) [2, 3] express a specific set of genes (“pluripotency genes”) that generate the gene regulatory network for pluripotency [4, 5]. Differentiation of hPSCs involves the suppression of these pluripotency genes such as POU5F1, SOX2, and NANOG, which maintain hPSCs in undifferentiated state, and the activation of early developmental genes, followed by the activation of tissue-specific genes [6–8]. Conversely, it is conceivable that forcibly altering the gene expression patterns from the pluripotent state to the cell-type-specific state would lead to the differentiation of hPSCs to the desired cell types in vitro.

The changes of gene expression patterns during myogenic differentiation have been well characterized. Several myogenic transcription factors (TFs) are identified as markers for specific stages in differentiation [9, 10]. The paired box transcription factors—Pax3 and Pax7—are specifically expressed in myogenic progenitors, such as satellite cells and myoblasts, and downregulated upon differentiation. The basic helix-loop-helix TFs—MyoD and Myf5—are activated in committed satellite cells and regulate skeletal muscle specification and differentiation. Using these markers as a guide, in vitro differentiation protocols have been developed: culturing hPSCs with other types of cells and in media supplemented with suitable growth factors [11, 12]. However, in most cases, the protocols require long-term, complicated steps, yet the efficiency of differentiation is rather low. To overcome these limitations, the forced ectopic expression of myogenic TFs in hPSCs has been effectively used [13–16]. Furthermore, recent studies have revealed that ectopic expression of epigenetic modifying factors such as a histone demethylase and an ATP-dependent remodeling factor significantly enhances the TF-mediated myogenic differentiation from hPSCs [17, 18].

In this review, we discuss the current methods for differentiating skeletal muscle cells from hPSCs through enforced epigenetic and transcriptional manipulation that can directly activate the myogenic gene expression program.

## 2. Forced Expression of TFs Leads to Direct Myogenic Differentiation of hPSCs

The first attempts to direct myogenic differentiation with forced expression of TFs have been performed using mouse ESCs (mESCs). Darabi et al. demonstrated that overexpressing Pax3 or Pax7 during the embryoid body (EB) formation of mESCs induces efficient myogenic differentiation [19–21]. When the mESC-derived myogenic progenitors are transplanted in dystrophic mice, these cells are engrafted in muscle and restore muscle function. Using the same approach, the authors subsequently showed that hESC- and hiPSC-derived mesodermal cells after PAX7 overexpression can give rise to myogenic progenitors with a high engraftment capacity in cardiotoxin-injured mouse muscle [14]. Furthermore, other studies have reported that forced expression of the myogenic regulator—MYOD1—also drives the myogenic differentiation of hESCs and hiPSCs. Goudenege et al. have demonstrated that the ectopic expression of MYOD1 converts mesenchymal cells derived from hESCs and hiPSCs (MB1-hPSCs) into engraftable myoblast-like cells [15]. Tedesco et al. have also reported efficient myogenic conversion of hiPSC-derived mesoangioblast-like progenitors (HIDEM) by the overexpression of MYOD1 [16].

In these experiments, the introduction of TFs was performed with lentiviral or adenoviral vectors, and their overexpression was not directly carried out in hESCs or hiPSCs but rather in mesodermal and mesenchymal-like cells derived from hESCs/hiPSCs. Indeed, the efficiency of myogenic differentiation is low when adenoviruses encoding MYOD1 are directly transduced to hESCs [15]. Albini et al. have also shown that lentivirus-mediated MYOD1 overexpression fails to induce myogenic conversion in hESCs, whereas comparable levels of MYOD1 expression efficiently induce myogenic differentiation from human fibroblast cells [18]. However, when a piggyBac vector system is used to express MYOD1, the direct myogenic conversion of hiPSCs can be successfully achieved [13], suggesting that a large amount of MYOD1 protein is required to activate skeletal myogenesis in hPSCs. These findings indicate that hPSCs have considerable resistance to MYOD1-mediated myogenic conversion.

## 3. Epigenetic Barrier of hPSCs against Differentiation

The functional activities of TFs require their physical access to the genome, which is tightly enclosed in chromatin structure consisting of DNA and histone complexes. Especially, the chromatin structure around the TF-binding sites plays critical roles in the regulation of gene expression [22–25]. The N-terminal tails of histone proteins are subject to various posttranslational modifications, including acetylation, methylation, phosphorylation, and ubiquitylation that result in changes of the chromatin structure [26–28].

**Figure 1:**
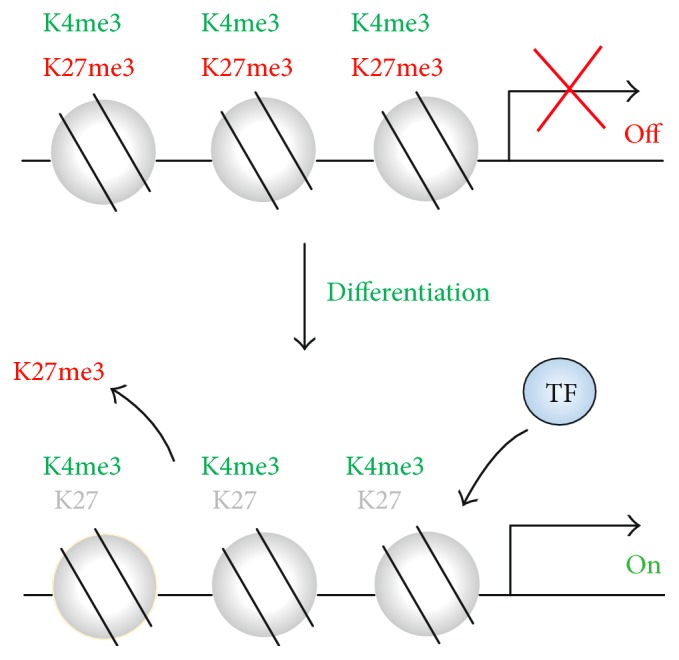
The chromatin regulation of developmental genes in PSCs. In PSCs, developmental genes are marked by bivalent domains containing both H3K4me3 and H3K27me3, which are associated with the transcriptional silencing in the undifferentiated state. The removal of H3K27me3 and the binding of transcription factors (TFs) allow rapid transcriptional activation upon differentiation stimuli.

Pluripotent ESCs/iPSCs possess unique chromatin signatures to be prepared for differentiation. In ESCs/iPSCs, lineage-affiliated genes are transcriptionally poised by “bivalent” histone modifications, consisting of H3 Lys-4 trimethylation (H3K4me3) and H3 Lys-27 trimethylation (H3K27me3) [29–32]. H3K4me3 is generally localized in the gene regulatory regions such as promoters and associated with transcriptional activation, whereas H3K27me3 is generally associated with inactive gene promoters [22]. However, in ESCs/iPSCs, both H3K4me3 and H3K27me3 are enriched in the promoter regions of genes associated with lineage differentiation (Figure 1). When differentiation is stimulated, ESCs/iPSCs initiate developmental programs by removing repressive H3K27me3 marks from lineage-affiliated gene promoters. Rapid gene expression can occur, because active H3K4me3 mark remains in those promoters. Loss of H3K27me3 by knocking out responsible histone enzymes results in derepression of developmental regulatory genes in mESCs and hESCs [33, 34]. Importantly, H3K27me3 represses the gene expression by impeding the binding and/or function of TFs and/or RNA polymerase [35, 36]. These results indicate that H3K27me3 functions as an “epigenetic barrier” against ESC/iPSC differentiation.

**Figure 2:**
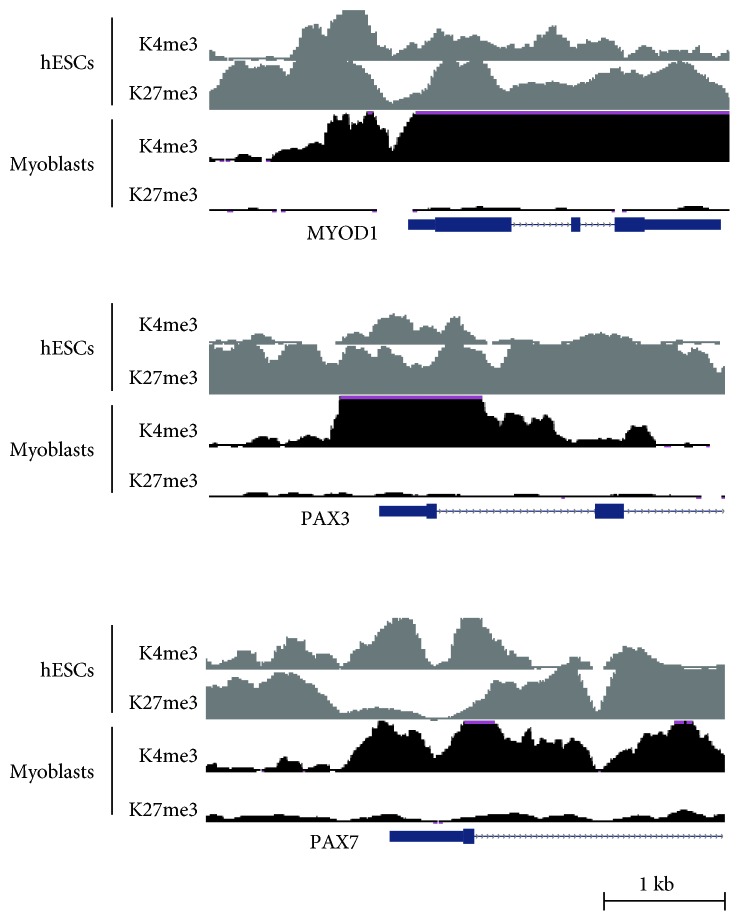
The states of H3K4me3 and H3K27me3 near myogenic genes in hESCs and myoblasts. The ChIP-sequencing peaks of H3K4me3 and H3K27me3 at the MYOD1, PAX3, and PAX7 genes are shown. The data were generated in the Bernstein laboratory [59].

In hESCs, more than 1500 genes are categorized as bivalent “H3K4/K27me3-modified genes” [30, 31, 37], which include myogenic regulatory genes, such as PAX3, PAX7, and MYOD1. Chromatin immunoprecipitation analysis revealed that both H3K4me3 and H3K27me3 are enriched in their promoters of hESCs, whereas only H3K4me3 is enriched in those of human myoblasts (Figure 2). These epigenetic states correspond to the expression states of the genes: they are repressed in hESCs and activated in myoblasts. These results suggest that the removal of H3K27me3 is crucial to switch the gene expression patterns of ESCs to those of muscle cells and to direct myogenic differentiation of ESCs/iPSCs.

## 4. Specific Enzymes Remove H3K27me3 during Differentiation

Histone methylation is dynamically regulated by two kinds of enzymes, histone methyltransferases and demethylases, which add and remove the histone lysine methylation, respectively. The addition of H3K27me3 is mediated by the Polycomb repressive complex containing the histone methyltransferase—EZH2—as the enzymatic subunit [38–40]. On the other hand, the removal of H3K27me3 is mediated by the Jumonji C (JmjC) domain containing demethylases—UTX and JMJD3 [41–43]. Knockdown and knockout experiments have shown that UTX and JMJD3 are required for the differentiation of mPSCs and hPSCs into endoderm, ectoderm, and mesoderm lineages [44–51]. Interestingly, although both UTX and JMJD3 are the specific demethylases of H3K27me3, their expression levels and patterns are completely different during the differentiation of hESCs. For instance, the expression of UTX is high in hESCs, whereas the expression of JMJD3 is quite low in these cells. Moreover, comparative transcriptome analysis between undifferentiated and differentiated ESCs has revealed that JMJD3 is significantly upregulated upon hESC differentiation into the three germ layers, whereas UTX is downregulated [17]. These findings suggest that JMJD3 upregulation is important for inducing H3K27me3 demethylation during the differentiation of hESCs.

## 5. Demethylation of H3K27me3 Facilitates MYOD1-Mediated Myogenic Differentiation

**Figure 3:**
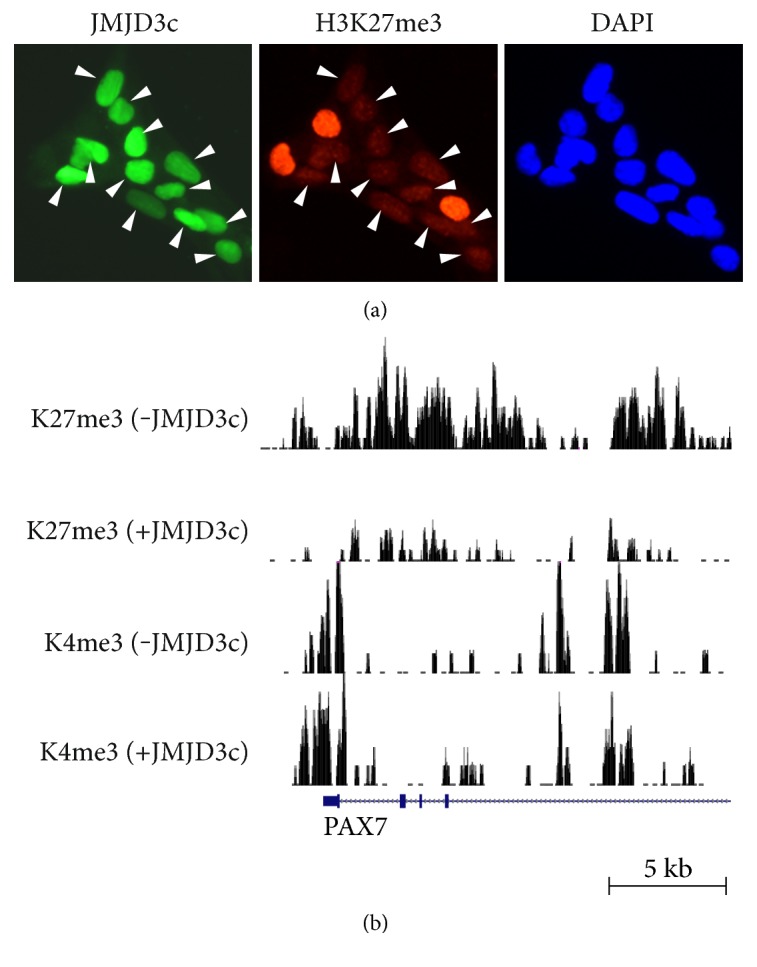
H3K27me3 demethylation in hESCs by forced expression of JMJD3c. (a) Immunostaining analysis showing the H3K27me3 demethylation in whole nuclei of hESCs by JMJD3c overexpression (arrows). (b) ChIP-sequencing analysis showing the significant reduction of H3K27me3 at the PAX7 gene in hESCs by JMJD3c overexpression. H3K4me3 enrichment remains at the PAX7 gene after JMJD3c overexpression.

Manipulating JMJD3 expression has the potential to change the epigenetic status of hPSCs toward differentiation. Indeed, Akiyama et al. have revealed that the forced expression of JMJD3 results in genome-wide demethylation of H3K27me3 in hESCs [17]. Furthermore, the overexpression of its C-terminal region containing catalytic JmjC domain (named “JMJD3c”) leads to more significant reduction of H3K27me3 compared to the full length of JMJD3 (Figure 3). When UTX, another H3K27 demethylase, is overexpressed instead of JMJD3, the demethylation of H3K27me3 does not occur. These results suggest that JMJD3 is a specific epigenetic modifier for generating the chromatin characteristics of differentiated cells.

The forced expression of JMJD3c enables the hESCs to upregulate developmental genes that are accompanied by H3K27me3 demethylation [17]. In this condition, genes associated with meso/endoderm differentiation are strongly activated compared to neuroectodermal genes. In addition to meso/endodermal TFs such as Brachyury (T) and SOX17, BMP and Wnt-signaling-related genes are also activated by the JMJD3c overexpression. As BMP and Wnt/*β*-catenin signaling is responsible for meso/endodermal differentiation [52, 53], ectoderm differentiation may be inhibited by JMJD3c overexpression through the mesoendoderm gene network.

JMJD3c overexpression also activates the PAX3 and PAX7 genes, but not MYF5 or MYOD1 [17], indicating that H3K27me3-deficient ES cells have a propensity to differentiate into myogenic progenitor cells. The chromatin states generated by JMJD3c overexpression may be similar to those of mesenchymal cells or mesoangioblast-like cells such as MB1-hPSCs or HIDEMs. Although MB1-hPSCs and HIDEMs are generated through signal transduction in response to changes in the culture conditions for differentiation, H3K27me3-deficient ES cells directly alter their gene expression patterns, resulting in exiting from the pluripotent state and upregulating developmental genes, even when the culture conditions for the hPSCs are not changed. Indeed, the activation of the PAX3 and PAX7 genes occurs within only a few days even in a medium that promotes the maintenance of an undifferentiated state.

The chromatin structure established in H3K27me3-deficient ES cells provides a suitable state for MYOD1-mediated myogenic differentiation. Akiyama et al. have shown that JMJD3c overexpression followed by MYOD1 overexpression significantly upregulates markers for skeletal muscle differentiation—MYOG, MEF2C, CKM, and SIX1 [17]. The myogenic gene expression program is quickly activated through the epigenetic changes. By 4 days after JMJD3c and MYOD1 overexpression, hESCs show expression patterns similar to the skeletal myotubes. JMJD3c cooperates with MYOD1 to activate the myogenic genes by changing the chromatin structure at their promoters. After JMJD3 and MYOD1 overexpression in hESCs, the MYOG and MEF2C promoters are enriched in active epigenetic marks—H3K4me3 and H3K27 acetylation.

## 6. Synthetic mRNA-Based Myogenic Differentiation of hPSCs

In previous studies, the overexpression of TFs was performed by viral or transposon vectors such as lentivirus, adenovirus, and piggyBac transposons. These vectors can effectively induce the expression of exogenous genes in hPSCs, but they have considerable limitations in terms of therapeutic applications: for example, possible insertional mutagenesis may occur due to random integration of the vectors into the host genome.

Synthetic mRNAs (synRNAs) encoding developmental regulator genes is one of the most promising approaches for directing the differentiation of hPSCs. This approach eliminates the risk of genomic DNA integration and insertional mutagenesis and is, thus, considered suitable for therapeutic applications. It has been shown that the transfection of synRNAs encoding reprogramming TFs into fibroblast cells can efficiently generate hiPSCs [54]. Furthermore, synRNAs encoding lineage-defining TFs such as Myod1, Hnf4a, and Ascl1 can differentiate mESCs into skeletal muscles, hepatocytes, and neurons, respectively [55]. However, in hPSCs, the efficiency of synRNA-mediated differentiation is low. Indeed, transfection of synRNA-encoding MYOD1 in hPSCs can generate only ~10% of myocyte-like cells. When hiPSCs were cultured in a fibroblast medium for 4 weeks and then transfected with synRNA-encoding MYOD1, ~40% of the cells became myogenic cells [54].

**Figure 4:**
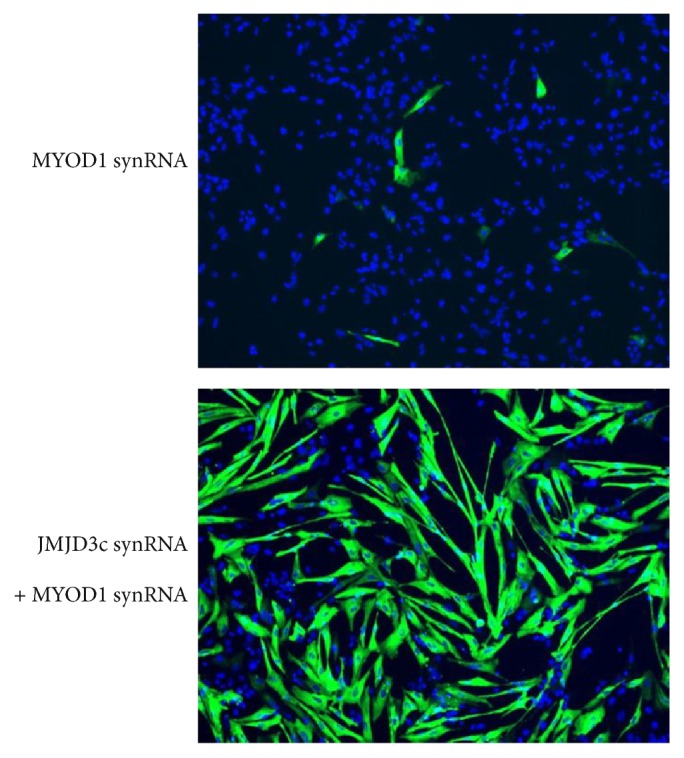
Efficient myogenic differentiation of hESCs by synRNA-mediated overexpression of JMJD3c and MYOD1. Transfection of synRNAs encoding JMJD3c and MYOD1 directly converts hESCs into MHC-positive myogenic cells for 5 days post differentiation. The differentiation efficiency was much higher in the JMJD3c/MYOD1-overexpressing hESCs compared with the MYOD1-overexpressing hESCs. MHC, green. DAPI, blue.

Akiyama et al. have demonstrated that transfection of JMJD3c-synRNAs prior to MYOD1-synRNAs dramatically increases the efficiency of myogenic differentiation of hPSCs [17]. The majority (>60%) of hESCs can be differentiated into myosin heavy chain- (MHC-) positive cells with myotube-like morphology in several days (Figure 4). By 4 days after transfection, some of the differentiated cells express a mature myogenic marker, creatine kinase-M, and possess the capacity for fusion with mouse C2C12 myoblast cells. These results suggest that the myotube-like cells induced by the JMJD3c and MYOD1 synRNAs have the potential to become mature skeletal muscles in vitro.

## 7. SWI/SWF Chromatin Remodeling Factor Enhances MYOD1-Mediated Myogenic Differentiation

A recent study showed that the SWI/SNF chromatin remodeling component BAF60C also promotes MYOD-mediated myogenic conversion in hESCs [18]. There are three variants of the BAF60 proteins, which are encoded by different genes: BAF60A (SMARCD1), BAF60B (SMARCD2), and BAF60C (SMARCD3). BAF60C is expressed in skeletal muscle cells but repressed in hESCs. The expression levels of one of the BAF60C isoforms, BAF60C2, significantly increase during embryoid body (EB) formation of hESCs. Albini et al. reported that sequential infection of BAF60C2 and MYOD1 lentiviruses enhances the activation of myogenic program in hESCs. BAF60C2 and MYOD1-overexpressing hESCs can be converted into MHC-positive cells with high efficiency (~60%) through changes of cell culture conditions: floating aggregates, followed by dissociation into single cells that are subsequently cultured in standard myogenic differentiation medium [18]. Infection of BAF60C2 lentivirus alone cannot activate the myogenic program in the absence of MYOD1. BAF60C2 facilitates the recruitment of MYOD1 and polymerase II to the target promoters by enhancing the chromatin accessibility. Interestingly, mesodermal genes such as Brachyury (T), MESOGENIN, and MESP1 are not upregulated by BAF60C2/MYOD1 overexpression, indicating that BAF60C2/MYOD1 can directly convert hESCs into the skeletal myogenic cells without the transition through the mesodermal stage. When BAF60C2/MYOD1-overexpressing hESCs are continuously cultured as floating clusters, they become contractile three-dimensional myospheres composed of skeletal myotubes.

## 8. Conclusion

In this review, we have provided an overview of the current status of skeletal muscle generation from hPSCs using epigenetic and transcriptional manipulation (Table 1). Direct differentiation of hPSCs hardly occurs with the ectopic expression of TFs alone. The forced introduction of epigenetic-modifying factors in hPSCs can facilitate the TF-mediated myogenic differentiation by bypassing or rapidly proceeding with the mesoderm stage. The combinatory approach using chromatin modifying factors and TFs will enhance the efficiency and robustness of RNA-based differentiation systems: an ideal method for generating footprint-free differentiated cells. Moreover, epigenetic variations are thought to be the main cause of significant variation in the differentiation capacities of different hiPSC lines [56–58]. Manipulating epigenetic states by using chromatin-modifying factors will allow the alteration of the epigenetic patterns of even low-potential hiPSC lines and improve their differentiation capacity.

## Conflicts of Interest

The authors declare that there is no conflict of interest regarding the publication of this paper.

## Figures and Tables

**Table 1:** Methods for the skeletal myogenic differentiation of hPSCs.

Overexpressed genes	Methods	Conditions	Maturation	Time	% MHC + cells	References
PAX7	Lentivirus	Doxycyclin-inducible overexpression in hPSC-derived mesodermal cells	Progenitors	2~3 weeks	>90%^∗∗^	[14]
MYOD1	Adenovirus	Infection in hPSC-derived mesenchymal cells	Mature cells	1~2 weeks	60%	[15]
MYOD1	Lentivirus	Tamoxyfen-inducible overexpression in mesoangioblast-like progenitors derived from hPSCs	Mature cells	3~4 weeks	>90%	[16]
MYOD1	piggyBac transposase	Doxycyclin-inducible overexpression in hPSCs	Mature cells	1~2 weeks^∗^	>90%	[13]
MYOD1	synRNA	Transfection of MYOD1 in hPSC-derived fibroblasts	Mature cells	5 weeks	40%	[54]
JMJD3c and MYOD1	synRNA	Transfection of JMJD3c followed by MYOD1 in hPSCs	Mature cells	5 days	60%	[17]
BAF60C and MYOD1	Lentivirus	Infection of BAF60C followed by MYOD1 in hESCs	Mature cells	1~2 weeks	60%	[18]

∗ includes the procedure for generating stable cell lines.

^∗∗^Differentiated cells from FACS-sorted PAX7-positive cells.
